# Multiplexed optical barcoding and sequencing for spatial omics

**DOI:** 10.1038/s41598-026-41186-y

**Published:** 2026-03-18

**Authors:** Aditya Venkatramani, Didar Ciftci, Khanh Pham, Limor Cohen, Leonardo Sepulveda, Christopher Li, Xiaowei Zhuang

**Affiliations:** Department of Chemistry and Chemical Biology, Department of Physics, Harvard University, Howard Hughes Medical Institute, Cambridge, MA 02138 USA

**Keywords:** Transcriptomics, Gene expression analysis, Transcriptomics, Transcriptomics

## Abstract

Spatial omics has brought a fundamental change in the way that we study cell and tissue biology in health and disease. Among various spatial omics methods, genome-scale imaging allows transcriptomic, 3D-genomic, and epigenomic profiling of individual cells with high spatial (subcellular) resolution but typically requires a preselection of targeted genes or genomic loci. On the other hand, spatially dependent barcoding of molecules followed by sequencing provides untargeted, genome-wide profiling but has a lower spatial resolution than imaging-based methods. Here, we report a spatial omics method that could potentially combine the power of the two approaches using optically controlled spatial barcoding followed by sequencing. Specifically, we utilize patterned light to encode the locations of molecules in tissues using oligonucleotide-based barcodes and then identify the barcoded molecular content, such as mRNAs, by sequencing. This optical barcoding method is designed with multiplexing and error-correction capability and achieved by a light-directed ligation chemistry that attaches distinct nucleic-acid sequences to the reverse transcribed cDNA products at different locations. As a proof of principle for this method, we demonstrated high-efficiency in situ light-directed ligation, spatially dependent barcoding with multiplexed light-controlled ligations at the single-cell level, and high-accuracy detection of spatially barcoded mRNAs in cells.

**Supplementary Information:**

The online version contains supplementary material available at 10.1038/s41598-026-41186-y.

## Introduction

Spatial omics provides a systematic approach to measuring expression profiles and molecular signatures of cells, identifying cell types and states in their native environment, and mapping spatial organizations and interactions of distinct cell types in tissues. Recent advances in spatial omics have enabled measurements of the transcriptome, 3D-genome, epigenome, and protein expression profiles of cells in tissues^1–3^. These measurements have generated detailed cell-type atlases of a variety of organs and whole organisms at various stages of development, revealed changes in gene expression patterns in disease states, and provided rich resources to study molecular interactions amongst different cell types^1,2,4^. The rapid pace of knowledge and hypothesis generation by this approach and the emergence of new demands have, in turn, provided an incentive to further develop and improve spatial omics methods.

Spatial omics methods fall broadly into two classes: approaches based on massively multiplexed imaging and approaches based on next-generation sequencing. Imaging-based methods allow simultaneous detection, quantification, and localization of thousands of RNA species and/or genomic loci, as well as dozens of proteins, with single-cell and subcellular resolution in three dimensions^2,3^. However, most imaging-based methods require the pre-selection of genes and genomic loci. On the other hand, sequencing-based methods do not require a pre-selection of targeted molecules and provide genome-wide information but have a lower spatial resolution than imaging-based methods. These sequencing-based methods generally rely on spatially dependent barcoding of molecules in samples using distinct DNA sequences, followed by extraction and sequencing of the molecular content to determine both the genetic identity and spatial locations of molecules^1,3^. Because such spatial barcoding occurs in two dimensions, either by capturing cellular RNAs on barcoded arrays or beads on a surface or by delivering barcodes into samples using microfluidics, these methods provide two-dimensional (2D) spatial omics information. The spatial resolution of these methods has been limited by the barcoding grid size (from sub-μm to tens of μm) and diffusion of molecular contents or barcodes (typical to a distance range comparable to the sample thickness, ~10 μm), thus making single-cell analysis challenging. The recently developed Slide-tag approach offers single-cell resolution but still lacks three-dimensional (3D) information^5^.

Light-controlled reactions provide an alternative approach to do spatially dependent barcoding. Because the spatial resolution of this approach is controlled by patterned light, it has the potential for 3D spatial omics with single-cell resolution. For example, the recently reported Light-seq method^6^ uses a photo-crosslinking chemistry to attach DNA barcodes directly to nucleic-acid contents within illuminated regions in cells and tissues down to the single-cell level. Another method, ZipSeq^7^, tags surface proteins with an antibody conjugated to a caged oligonucleotide sequence and introduces spatial barcodes through photo-uncaging and hybridizing a barcode to the uncaged portion of the oligonucleotide. To date, these light-controlled barcoding approaches have been used to provide RNA or protein expression profiling of a small number of spatial regions in the sample. For example, as a proof-of-principle, three different barcodes have been generated for distinguishing three spatial regions in the Light-seq study^6^. It remains to be demonstrated whether the photochemical reactions used in these methods allow a high degree of spatial multiplexing (namely omics measurements of a large number of spatial regions per sample).

Here, we report MOLseq (Multiplexed optically-controlled ligation followed by Sequencing), a light-directed spatial omics technique that uses a different photochemical approach to generate spatial barcodes as compared to Light-seq and Zipseq. MOLseq uses an optically controlled ligation chemistry to iteratively add short oligonucleotide sequences at specific locations in samples as spatial barcodes, followed by extraction and sequencing of barcoded molecular contents of samples for genome-wide profiling. MOLseq constructs barcodes in a combinatorial manner over multiple rounds of ligation, thus providing a scalable approach for barcoding where the barcode diversity grows exponentially with the number of ligation rounds. Barcodes capable of error detection and correction could be used to further increase measurement accuracy. The spatial positions of barcodes are determined by light and, hence, has a potential to offer high spatial resolution. We showed that MOLseq can be used to generate a diverse set of barcodes in situ and control the spatial positions of barcodes down to the single-cell level. We also demonstrated proof-of-principle spatially resolved, untargeted transcriptomic profiling of cells with a cell culture model.

## Results

### Concept of MOLseq for spatial omics

**Fig. 1:**
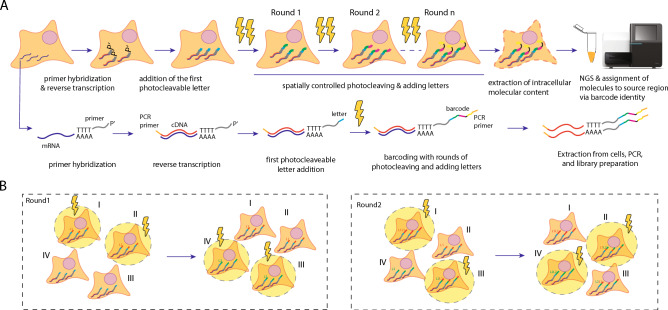
Multiplexed optically controlled barcoding for spatial omics. (**A**) MOLseq experimental workflow illustrated in a cell. MOLseq encodes the spatial position of molecules in the form of oligonucleotide-based barcodes. First, a DNA primer is hybridized to the molecules of interest, such as a poly-T primer targeting the mRNA molecules, followed by in situ reverse transcription to generate cDNAs and template switching to introduce a PCR primer at the 3’ end. We then build a barcode sequence at the 5’ end of the cDNAs in a spatially dependent manner using light control, with each UV illumination adding one letter to the barcode. A PCR primer is then added to the 5’-end after the barcode using the same light-controlled process. Cells are then lysed, and the extracted material is amplified by PCR, processed into a sequencing library, and then sequenced. The barcode sequences allow the assignment of identified molecules to their spatial position, creating a spatially resolved omics map. (**B**) Multiplexed generation of spatial barcodes using patterned light. Barcodes are generated by the iterative addition of unique oligonucleotide sequences to the primer in regions illuminated by light. Here, we depict two rounds. Each round completes the addition of one letter to the barcode, either L_1_ or L_2_. Regions I and II, which have the letter L_1_ in the first digit position, are illuminated at the same time, followed by the illumination of regions III and IV and the addition of the letter L_2_ in their first digit. This completes the first round, and we add the second letter to the barcodes in a similar manner in the second round. In this manner, barcode diversity grows exponentially with the number of rounds, conferring scalability and multiplexity to the design.

As the first step in MOLseq, we target specific groups of molecules in a sample using a DNA primer, the sequence of which can target, for example, the polyA tail of mRNA, other cellular RNAs, or oligo-conjugated antibodies for proteins. In this work, we target the polyA tails of mRNAs by hybridizing them with a poly-T primer, followed by reverse transcription in situ to create cDNAs. We then attach unique oligonucleotide sequences (barcodes) to the cDNA products at different locations in the sample. The spatially barcoded cDNAs are then extracted and sequenced to determine the genetic identities and spatial locations of the mRNAs (Fig. 1A). To multiplex barcoding, we consecutively attach multiple oligonucleotide sequences to cDNAs at each location in a light-directed manner, each oligonucleotide sequence corresponding to a letter in the barcode and each light illumination event adding only one letter. The order of the letters then determines the barcode corresponding to each spatial location.

Figure 1B illustrates an example of such a barcoding process. Suppose we have two letters, L_1_ and L_2,_ and aim to barcode four regions: region I = L_1_L_1_, region II = L_1_L_2_, region III = L_2_L_1_, and region IV = L_2_L_2_. In the first round, regions I and II are illuminated with light and the letter L_1_ is added. Then, regions III and IV are illuminated with light and the letter L_2_ is added. In the second round, we illuminate regions I and III and add an additional letter L_1_ to these regions, followed by illuminating regions II and IV and adding an additional letter L_2_ to these regions to finish the barcoding process. This example illustrates the multiplexity and scalability of this barcoding approach: with *m* letters and *n* rounds, we can generate *m*^*n*^ unique barcodes in *m* x *n* letter-ligation steps (i.e., *m* steps for each of the *n* rounds; spatial locations encoded with the same letter in the same round can be illuminated simultaneously). Moreover, the barcoded regions do not have to cover the whole sample and can be chosen based on interest.

At the end of the barcoding process, each cDNA contains both the spatial barcode and transcript identities. We then extract cDNAs from cells and process them for next-generation sequencing (NGS) (Fig. 1A). A variety of NGS platforms are available, which offer different sequencing depth and read length options. An optional 5’ enrichment step can be implemented during library preparation for platforms with short read length or limited depth (Supplementary Figure 1).

### Light-controlled in situ barcode generation

**Fig. 2:**
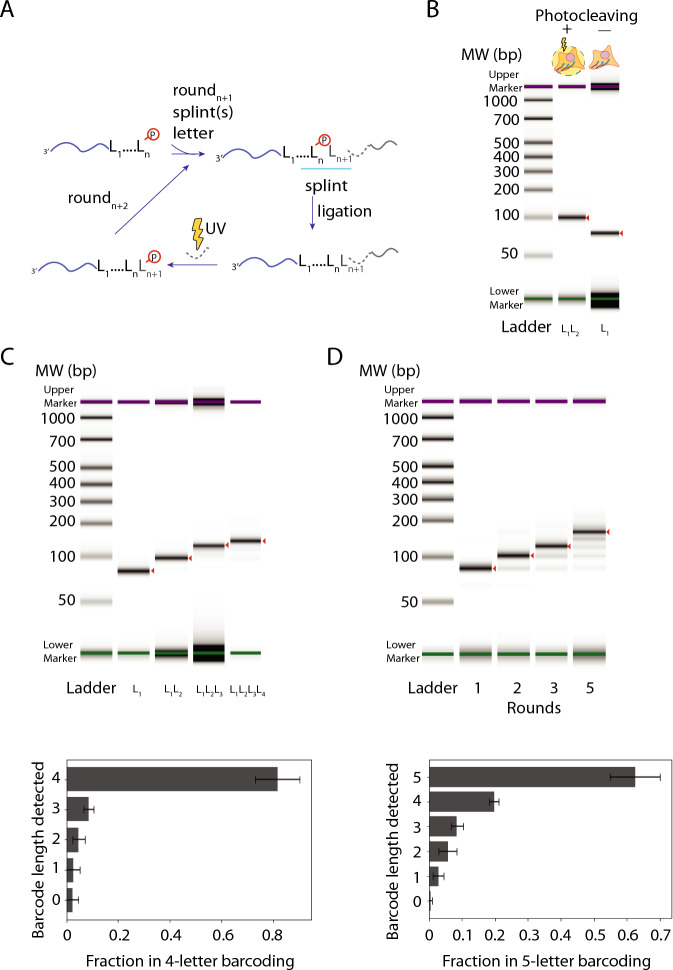
Iterative light-controlled ligations of letter-representing DNA sequences with a photocleavable spacer. (**A**) A barcode is constructed from a sequence of letters L_1_, L_2_, ..., L_n_, L_n+1_, …. The existing letter on the barcode has a phosphate group at the 5’ end. The next letter with a photocleavable spacer (PC, shown in dashed lines) and a splint are added to enable the ligation reaction, extending the barcode by one letter. When exposed to UV light, the PC gets cleaved, exposing a free 5’ phosphate group, which allows for the next round of ligation. (**B**) Agilent 2200 TapeStation gel image of the extracted material from two samples of cultured U2OS cells, one of which has been exposed to UV light after the first round of ligation (lane labeled L_1_L_2_) and the other one which has not (lane labeled L_1_), preventing the addition of the second letter. The left lane is the molecular weight (MW) standard labeled as “Ladder”. (**C**) Top, TapeStation gel image of sequential ligations of letters showing the construction of 1, 2, 3, and 4-letter barcodes (lanes labeled L_1_, L_1_L_2_, L_1_L_2_L_4_, and L_1_L_2_L_3_L_4_, respectively). Bottom, Quantification of the 4-letter barcode construction case. Bar plots show means ± SD (*N* = 3 replicates). (**D**) To demonstrate barcode multiplexity, we use all four letters with all the necessary splints for each round of letter addition to create 1, 2, 3, and 5-letter barcodes. Top, TapeStation gel image of such multiplexed barcoding cases to create 1, 2, 3, and 5-letter barcodes. Bottom, Quantification of the 5-letter barcode construction case. All experiments were performed in cultured U2OS cells. All gel images are generated by Agilent 2200 TapeStation software, as a representative example from three replicates, where the expected length barcode is marked with a red arrowhead. Bar plots were derived from plotting the normalized intensity of each peak from the electropherogram for that sample.

We generate light-controlled barcodes through recursive ligations of letter-representing DNA sequences with a photocleavable spacer (PC) linked to another oligonucleotide sequence (Fig. 2A, Supplementary Figure 2)^8,9^. The process begins with a primer that has a 5’ phosphate group, to which we ligate a letter using a ligase enzyme. A splint is chosen to facilitate the ligation, with a complementary sequence to a part of the current barcode sequence and a part of the incoming letter. The ligation product is then exposed to UV light to photocleave the PC and expose a 5’ phosphate group, allowing the next round of ligation to occur, as illustrated in Fig. 2A. If the letter is not photocleaved, there is no 5’ phosphate group, preventing further ligation. In a proof-of-principle experiment with two rounds of ligations (one with letter L_1_ and the other with letter L_2_) performed on cultured Human Osteosarcoma (U2OS) cells, in which one of the samples was exposed to UV after the first round of ligation of letter L_1_ and the other sample was not, we extracted and analyzed the barcoding products by capillary electrophoresis using DNA ScreenTape assay and represented the electropherogram results in a gel format generated by the TapeStation software. We indeed observed ligation of letter L_2_ only to the sample that was exposed to the UV light, but not to the other sample (Fig. 2B).

Next, we demonstrated that this process can be used to continuously add multiple letters with high efficiency by generating 1, 2, 3, and 4-letter long barcodes (each letter 20 bases long) in U2OS (Fig. 2C). To facilitate quantification of the barcoding products by TapeStation, the poly-T primer was not reverse transcribed to preserve the original sequence length and had a PCR primer at the 3’ end to allow PCR amplification. After the light-controlled ligation steps, the barcoded primer was extracted, and a second strand was generated through one cycle of PCR followed by purification or through hybridization with a complementary oligonucleotide (Methods). The products were analyzed by TapeStation, and we observed both the correct size band corresponding to products with the expected number of barcode letters and smaller bands arising from inefficient photocleaving or ligation. From the fraction of products with the expected number of barcode letters in the 4-letter barcoding case, we estimated the efficiency of each round of letter addition to be 94.9 ± 2.6% (mean ± SD, *N* = 3 replicates, Fig. 2C), through a simple model assuming each letter can be added independently from the previous round of letter addition. For example, a 4-letter barcode requires four independent successive rounds of photocleaving and ligation reactions, meaning that the single letter addition efficiency (*p*) can be estimated from the equation *f* = *p*^4^, where *f* is the fraction of primers observed to carry 4-letter barcodes.

When generating distinct barcodes in parallel, we need to add a letter to any possible combination of existing letters on the cDNAs to ensure that this process is multiplexable. To achieve this, we could use a pool of splints, each of which has complementary sequences to the incoming letter and one of the previously added letters. Figure 2D demonstrates barcode generation by ligating four different letters over 1, 2, 3, and 5 rounds with a pool of all possible splints that could facilitate ligation of all 4 incoming letters and all prior letters (a total of 4 x 4 splint sequences). Similar to the efficiency estimation in the single barcode case described above, we estimated the efficiency of letter addition per round to be 90.9 ± 2.3% (mean ± SD, *N* = 3 replicates, Fig. 2D) for this 5-letter barcoding process. We then sequenced the 5-letter-long barcode and detected 756 ± 24 (mean ± SD, N = 3 replicates) unique barcodes. This number is less than the expected number (4^5^ = 1024) of all possible barcodes. We reason that when all 4 letters are present in every round, different letters may compete with each other when the ligation efficiency is not the same for all letters, which could result in a reduced number of barcodes generated. Hence, the barcode diversity could potentially be higher in the actual MOLseq barcoding process, where only one incoming letter is present for ligation at any time without potential competition from other letters.

### Light-controlled barcoding in a spatially resolved manner

**Fig. 3:**
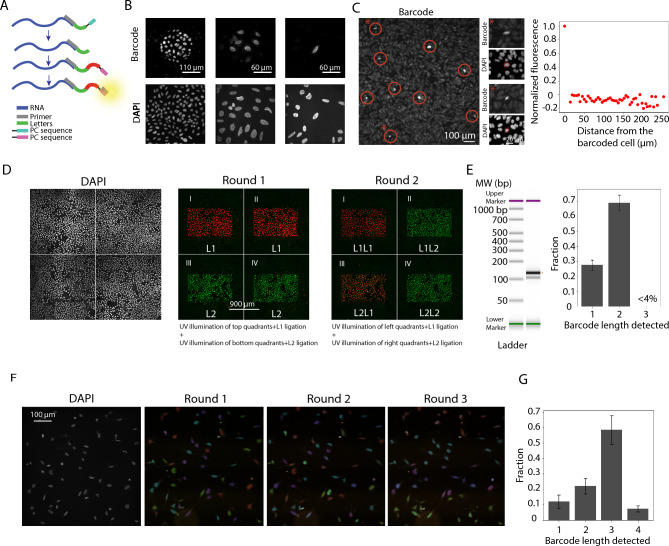
Spatially controlled barcode generation via patterned light. (**A**) Detection scheme of letters in the barcode. A primer is hybridized to mRNA, and a letter with a PC is ligated to the primer. DMD-controlled light photocleaves the PC and the next letter is added. Letter ligation is detected through FISH. (**B**) Spatially controlled letter addition to regions of 220 µm, 90 µm, and 20 µm in diameter. FISH signal of barcoded product and 4’,6-diamidino-2-phenylindole (DAPI) signal are shown in top and bottom panels, respectively. (**C**) Left: Distance dependence of off-target photocleaving and ligation rates. Barcoded cells are circled. Examples marked with an asterisk are enlarged in the right panels, with the top/bottom panels showing barcode FISH signal and DAPI signal, respectively. Right: Normalized fluorescence of the neighboring cells up to 250 µm away from the barcoded cells is averaged. (**D**) Construction of unique barcodes in four different sample regions from two letters detected with Alexa647 (red) and ATTO565 (green) labeled FISH probes. DAPI signal is shown in the left panel. (**E**) Left: TapeStation gel image of barcoded material in (D). Left lane: molecular weight standard. Right lane: barcoded material. Bands with the expected barcode lengths are marked by red arrowhead. Right: Quantification of relative intensities of products. 3-letter barcode product was undetectable in the TapeStation gel image and quantification trace, suggesting that such product was absent. Using a multi-Gaussian fit, we estimate the upper bound of 3-letter barcode to be <4%. (**F**) Construction of 64 unique barcodes using letters L_1_, L_2,_ L_3,_ L_4_ over three rounds of barcoding, imaged with two-color FISH (Alexa 647 and ATTO565) in two hybridizations. Letters are pseudo-colored as yellow-green, purple-blue, cyan, and red. (**G**) Quantification of relative intensities of products. All experiments were performed in cultured U2OS cells. Images are one representative example from three replicates. Bar plots were derived from fitting a sum of Gaussians to the TapeStation electropherogram.

We generate spatially selective barcodes by directing patterned UV light onto the sample using a Digital Micromirror Device (DMD). Using poly-T primers attached to mRNAs in cultured U2OS cells as a model system, we demonstrated spatially controlled photocleaving and ligation on selected sample areas ranging from hundreds of microns down to a single cell by using fluorescently labeled FISH probes complementary to ligated letters to detect the barcodes (Fig. 3A, B).

In contrast to experiments presented in Fig. 2, the use of spatially controlled photocleaving introduces a possibility of off-target photocleavage, which can subsequently lead to barcode ligation. We quantified the distance dependence of off-target photocleaving and ligation rate by plating cells at a near confluent cell density and measuring barcode signal intensity (measured by FISH) in neighboring cells as a function of the distance from the barcoded cell. We observed that even the nearest neighbor cells, which were on average 15 µm away from the barcoded cell, had close to zero FISH signal upon background subtraction, suggesting a minimal distance-dependent off-target photocleaving and ligation rate (Fig. 3C).

Next, to demonstrate the ability to construct diverse barcodes in different spatial regions, we generated four distinct barcodes in four distinct spatial locations in the cell culture using two letters, L_1_ and L_2_, which can be detected using two differently colored fluorescent probes (false-colored red and green for L_1_ and L_2_, respectively, in Fig. 3D). In the first round, we added the letter L_1_ to two regions (regions I and II) and the letter L_2_ to two other regions (regions III and IV). In the next round, we again ligated L_1_ and L_2_, but to different region combinations (L_1_ to regions I and III, and L_2_ to regions II and IV). After each round, we detected the ligation product by FISH probes that were complementary to the sequence downstream of the PC moiety (5’ end) in the letter-representing oligonucleotide (Fig. 3A). Indeed, FISH imaging verified the successful ligation of letters L_1_ and L_2_ in the designed order in four sample regions in a multiplexed manner (Fig. 3D).

As further validation, we ligated a PCR primer to the barcodes in the four regions, extracted the barcoded products, PCR-amplified them, and analyzed them by TapeStation. To ensure that the PCR primers were attached primarily to the barcodes, we used splints that correspond only to letters in the barcode. The TapeStation electropherogram result did not show any peak (or band in the gel image) that correspond to a 3-letter barcode, suggesting the absence of such a product. Nonetheless, we estimated an upper bound of the fraction of 3-letter long barcodes that could result from potential off-target photocleaving and ligations by multi-Gaussian fitting (see Methods). We observed that the majority (69.2 ± 5.0%, mean ± SD, *N* = 3 replicates) of products had a 2-letter long barcode, with only a small fraction (<30%) having 1-letter barcode, and that the upper bound of the fraction with 3-letter barcode was estimated to be 3.5% (Fig. 3E).

To quantify the off-target letter addition rate in these experiments, we estimated the per-round on-target letter addition probability, *d*, and the per-round off-target letter addition probability, *c*, from the observed fractions of 2- and 3-letter long barcodes using a combinatorial probability model that accounts for all possible ways a barcode of certain length can be generated from various on-target and off-target letter addition combinations (see Methods). Using this approach, we estimated the per-round on-target letter addition probability, *d,* to be 84.5 ± 4.1%, which is reduced compared to the experiments in Fig. 2, likely due to the more conservative illumination intensity used when performing spatially controlled experiments. The probability of adding a letter through off-target events*, c*, was calculated to be 2.5 ± 1.8%.

We notice that the signal in region III of Round 2 deviated from red, due to the presence of some green signal (Fig. 3D). We think this is unlikely due to off-target letter addition of L_2_ for two reasons: (i) such off-target letter addition was not observed in Round 1 or other regions in Round 2 (Fig. 3D); (ii) off-target letter addition would generate barcodes with >2 letters, and such products were essentially absent (Fig. 3E). We think that the observed mixture of red and green signals in region III in Round 2 is most likely a result of ATTO565 conjugated probes not being fully washed out after the first round.

We further extended our barcoding experiments to more distinct regions and demonstrated barcoding at the single-cell level by adding distinct barcodes to 64 individual cells (Fig. 3F). To this end, we segmented single cells (nuclei of individual cells in this case) from DAPI images, barcoded 64 of them using four letters, L_1_, L_2,_ L_3,_ L_4_, over three barcoding rounds, and detected the ligated letters in each round by using FISH probes targeting a region in the freshly ligated letters downstream of the PC moiety (Fig. 3F). We then validated the barcode length by ligating a PCR primer to the barcodes and analyzing the extracted products by TapeStation after amplification (Fig. 3G). We observed that the majority of the products (58.3 ± 9.4%, mean ± SD, *N* = 3 replicates) had 3-letter long barcodes, but a small fraction (7.4 ± 2.0%, mean ± SD, *N* = 3 replicates) had 4-letter long barcodes, likely due to off-target letter addition (Fig. 3G). Using the same model as described above, we estimated the per-round on-target letter addition probability, *d,* to be 85.4 ± 5.6% and the probability of per-round off-target letter addition *, c*, to be 1.4 ± 0.7%, similar to the rates derived from experiments shown in Fig. 3D, E. The median distance between neighboring cells is 44 um in this experiment (Supplementary Figure 3).

We note that these experiments retained barcode species generated through all possible off-target events, caused not only by barcode-writing light when barcoding neighboring regions, but also by stray or scattered light, background ambient light, and possibly a small level of light-independent photocleavage and ligation. Thus, these experiments provided a more comprehensive estimation of off-target letter addition probability, as compared to measurements shown in Fig. 3C, which were instead used to estimate only the distance dependence of off-target events due to barcode writing light.

### Spatially resolved transcriptomics by light-controlled barcoding

**Fig. 4:**
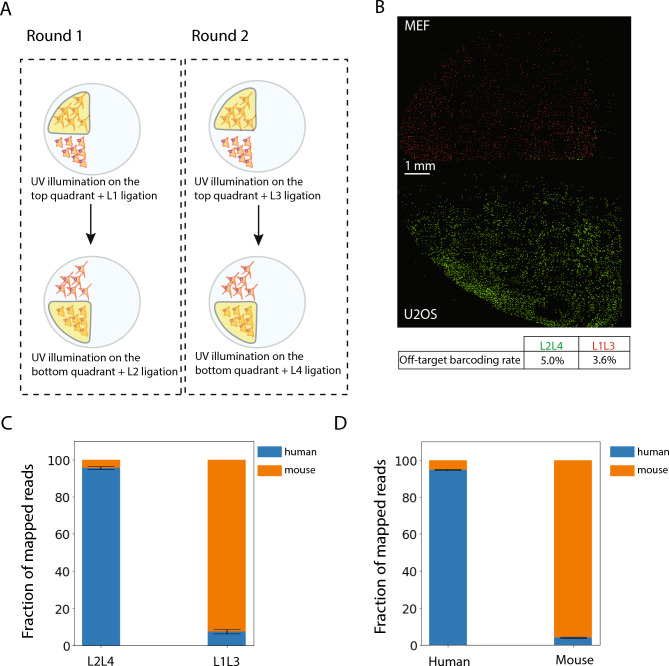
Spatially resolved transcriptomics by light-controlled barcoding with MOLseq. (**A**). Mouse and human cell lines are plated on the top and bottom sides of the sample, respectively, and four letters are used to construct two unique barcodes encoding the two regions (L_1_L_3_ for the top region) and (L_2_L_4_ for the bottom region). (**B**) Image of the U2OS and MEF cells hybridized with FISH probes complementary to half of each letter in the barcode sequence. FISH probes for the barcode L_2_L_4_ are labeled with Alexa647 (green), and FISH probes for barcode L_1_L_3_ are labeled with ATTO565 (red). The image is one representative example from three replicate experiments. The table at the bottom shows the quantification of the off-target barcoding rate, calculated as the ratio of the number of cells with red or green fluorescence on the untargeted quadrant to all of the red or green cells, respectively. On average 5.0% and 3.6% (*N* = 2 replicates) of the barcode cells were located in the off-target regions for the L2L4 and L1L3 barcodes, respectively. (**C**) Fraction of the sequencing reads containing the specified spatial barcodes that are mapped to the human and mouse genome. For the barcode L_2_L_4_, 95.7 ± 0.9% (means ± SD, *N* = 3 replicates) of all mapped reads are human, and for the barcode L_1_L_3_, 92.7 ± 1.2% (means ± SD, *N* = 3 replicates) all mapped reads are mouse. (**D**) Species distribution of reads generated from U2OS and MEF cells cultured in physically separate chambers and processed separately until the sequencing stage. The cross-species mapping rate is 4.7 ± 0.4% (means ± SD, *N* = 3 replicates) in this case.

As a proof-of-principle validation of our light-directed barcoding approach to provide spatially dependent transcriptomic profiling, we performed a mixed-species experiment using Human U2OS and Mouse Embryonic Fibroblast (MEF) cells, plated in two separate regions of the same coverslip (Supplementary Figure 4). This approach allowed us to create distinct but adjacent regions of cells from different species. We employed the full MOLseq process to barcode and sequence this sample (Fig. 4A). We first hybridized poly-T primers to the sample, performed reverse transcription to extend the primers, and then barcoded cDNA products in two different regions, each with a unique 2-letter barcode, namely L_2_L_4_ for the U2OS cells on the bottom side and L_1_L_3_ for the MEF cells on the top side of the sample. We note that building barcodes with more than one letter that is distinct between the two regions introduces error-detection capability and increases the accuracy of spatial barcoding. Approximately 3,000 cells were barcoded in each species. We confirmed the generation of 2-letter barcodes in a region-dependent manner by detecting the presence of the expected barcodes using FISH with differently colored probes for different barcodes (Fig. 4B and Supplementary Figure 4). We found that each barcode’s fluorescent signal was highly specific to its targeted region, where only 5.0% of the cells containing the L2L4 barcode and 3.6% of the cells containing the L1L3 barcode were observed in off-target regions (percentages are mean from *N* = 2 replicates). Because the quadrant separation distance was greater than 0.5 mm, the off-target letter addition rate from barcode writing light should be negligible. Given that incorrectly colored cells in the off-target region were largely observed on the edge of the region, it is possible that they were due to cell migration during barrier removal and subsequent pipetting.

We then extracted the cDNA content, enriched for the 5’ end containing the barcode and part of the transcript sequence (Supplementary Figure 1), and performed short read sequencing with paired-end reads, generating a total of ~70 million reads per replicate (*N* = 3 replicates). The reads were mapped to mouse and human genomes, and intronic and intergenic reads were filtered out. We detected ~9500 human genes and ~6300 mouse genes on average (*N* = 3 replicates). We note that in this proof-of-principle demonstration, our sequencing depth was relatively low compared to typical RNA-seq analysis and we anticipate that more genes could be detected with increasing sequencing depth. Nonetheless, these results were sufficient for us to determine the spatial accuracy of the measurements. For the reads containing the barcode L_2_L_4_, relative fractions of reads mapped to human and mouse species were 95.7 ± 0.9% and 4.3 ± 0.9% (mean ± SD, *N* = 3 replicates). For the reads containing the barcode L_1_L_3_, relative fractions mapped to human and mouse species were 7.3 ± 1.2% and 92.7 ± 1.2% (mean ± SD, *N* = 3 replicates) (Fig. 4C).

For the reason stated above, the ~4-7% misidentification rate is unlikely to be solely caused by off-target barcoding. We therefore reasoned that sequence homology between mouse and human could also contribute to the misidentification rate. Indeed, when we cultured U2OS and MEF cells in physically separate chambers and processed them separately until the sequencing stage, we observed a 4.7 ± 0.4% cross-species mapping rate (mean ± SD, *N* = 3 replicates) (Fig. 4D), presumably due to a certain level of homology existing between mouse and human transcriptomes, consistent with results from previous studies^10^. Hence, the observed small mis-classification rate in the experiments described in Fig. 4C likely arose primarily from the homology between mouse and human transcriptomes, suggesting a high accuracy of MOLseq in this measurement.

## Discussion

In this work, we developed MOLseq as an approach that brings together the power of optical control and sequencing for spatial omics in a multiplexed and scalable manner. The iterative in situ barcode construction, using light-directed cleavage and ligation of oligonucleotides, can generate a diversity of barcodes, which has a potential to allow high-throughput spatial omics measurements across a wide range of length scales from tissue regions to single cells. Thanks to the flexibility and versatility of light patterning, the resolution and number of targeted spatial regions can be adjusted based on experimental needs.

We demonstrated in cultured cells in situ light-controlled DNA cleavage and ligations with up to ~90% efficiency per ligation round. Using a pooled mixture of splint sequences to allow any letter-representing oligonucleotide sequence to be added to desired locations independent of the previously added letters, we can construct a high diversity of barcodes in a time-efficient manner. Indeed, we generated ~750 unique barcodes with only 5 rounds of ligations using pooled mixtures of barcode letters and ligation splints, demonstrating that this multiplexed, light-controlled ligation is a viable approach for scalable spatial barcode generation. The multi-letter-based barcode design not only allows for scalability but also provides error detection and correction capability. Using a DMD to select photocleaving and ligation regions at various length scales, we demonstrated optically controlled barcoding in regions ranging from millimeters to single cells (~20 microns) and obtained high spatial specificity. Combining in situ optically controlled barcoding with sequencing, we demonstrated spatially resolved transcriptomic profiling of cells. The data presented here focuses on mRNA measurements in cells, but MOLseq barcoding can be adapted to measure other molecular species, such as detecting protein molecules by oligonucleotide-conjugated antibodies^11–14^.

MOLseq also has limitations in its current form. Since it relies on photocleaving by patterned light and subsequent ligation for spatial selectivity, off-target photocleaving from the tails of patterned UV light, stray light, or background light-independent cleavage can introduce errors, leading to barcode crosstalk between spatial regions. For example, in our single-cell barcoding experiments, we observed a small level of off-target letter addition. This error could be reduced further by using error-robust barcodes capable of error detection and correction. Other challenges could also be present for achieving high spatial measurement. For example, the amount of RNAs present in a single cell is small, and hence high efficiency of barcode generation and RNA extraction would be required for analysis at the single-cell level. An important future development is to overcome these challenges to allow spatial omics profiling with high spatial resolution at the single-cell level.

In addition, barcode diversity generated by MOLseq is limited by photocleavage and ligation efficiency. With the current ~90% efficiency per ligation round, it would be reasonable to limit the number of ligation rounds (*n*) to ~5, resulting in ~60% of molecules with full-length barcodes, which should allow reasonably accurate sequencing analysis. In order to generate a diversity of 10^5^ barcodes (i.e., measure 100,000 spatial regions simultaneously), we will need the number of distinct letters (*m*) to be 10 because the barcode diversity is equal to *m*^*n*^. Since we construct the barcodes by ligating one letter at a time, this would require 10 ligation steps per round, and hence 5 x 10 = 50 total ligation steps to generate the barcodes, which will take ~3 days based on our current protocol. The pre-barcoding steps of primer hybridization and reverse transcription take a day each, together with the post-barcoding steps of extraction and library preparation, the entire protocol will take ~6 days, after which the product can be sent out for sequencing. An increase in *n* could rapidly increase barcode diversity with a minimal effect on the total barcode generation time if we keep *m* x *n* constant by reducing *m* accordingly (for example, 5^10^ is approximately equal to 10 million). However, with ~90% ligation efficiency per round, only ~30% of molecules will have full-length barcodes after 10 rounds of ligation. We could use barcodes capable of error detection and correction to effectively increase this overall barcoding efficiency. For example, using a repeat code in which each letter appears twice and correctly assigning the barcode by the presence of at least one of the two repeats, we could effectively increase the per letter efficiency from 90% to 99% and hence increase the fraction of molecules with full-length barcodes. This approach would, however, increase the total amount of time needed to generate barcodes. Thus, another important future development is to improve the ligation efficiency, hence increasing time efficiency and diversity in barcode generation.

Light-directed barcoding methods for spatial omics have both advantages and limitations compared to existing imaging-based and sequencing-based spatial omics approaches. Unlike imaging-based methods, which typically require a pre-selection of genes, light-based barcoding enables untargeted, genome-wide profiling similar to other sequencing-based methods. On the other hand, imaging-based approaches can achieve very high spatial resolution in 3D, capturing subcellular features. Although light-directed barcoding methods have a potential to achieve spatial omics with high spatial resolution, achieving sub-cellular resolution could still be difficult. As we reported this work in bioRxiv^15^, two parallel studies on the development of the light-controlled ligation approaches for spatially resolved transcriptomic profiling were reported in bioRxiv at around the same time^16,17^. One of these studies demonstrated the ability to characterize the transcriptome of subcellular compartments, but one compartment at a time without multiplexing^17^. Future development may allow multiplexed detection of multiple subcellular compartments, but this approach could be limited by the off-target effect of light-directed barcoding in neighboring subcellular regions. In addition, although light-directed barcoding methods could potentially achieve spatial omics in 3D using 3D light pattern generation, it will be technically more challenging than 3D profiling using imaging-based approaches. Moreover, the transcripts need to be extracted for sequencing, and because of low capture efficiency, light-directed barcoding methods for spatial transcriptomics likely will have lower detection efficiency (and hence effectively also lower spatial resolution) than imaging-based spatial transcriptomics approaches. Compared to other sequencing-based approaches without light control, light-directed barcoding methods have a potential for higher spatial resolution. Moreover, light directed barcoding methods also offer the potential to target rare cell types, for example those identified by morphology or immunostaining signatures, by precisely directing barcoding to regions of interest. However, the light-directed reaction steps involved in these methods may decrease barcoding efficiency. Scaling to a large number of spatial regions could also be more challenging than sequencing-based approaches without light control.

In their current state, light-directed barcoding approaches for spatial omics are much less developed than either imaging-based or non-light-directed sequencing-based approaches. In particular, experimentally demonstrated number of spatial regions that can be profiled per sample by light-directed barcoding approaches is much smaller than the other two categories of approaches. Future development of these approaches, including further improvement of the photochemistry used in MOLseq, LightSeq and ZipSeq, development of other photochemical schemes, as well as development of precise and scalable light patterning and high efficiency extraction of barcoded materials from the sample, are needed to realize the full potential of light-directed barcoding approaches for spatial omics.

## Methods

### Sample preparation of cells

We used Human Osteosarcoma (U2OS) or Mouse Embryo Fibroblast (MEF) in our experiments purchased from ATCC (catalog numbers HTB-96 and CRL-2991 respectively). The cells were cultured and plated onto Ibidi µ-Slide VI 0.4 lanes for barcoding experiments without reverse transcription and sequencing and Ibidi Culture-Insert 4 Well in µ-Dish 35 mm for the co-culture and MOLseq experiment. The Ibidi chambers were incubated with Poly-D-Lysine solution (0.1 mg/ml, Thermo Fisher, A3890401) for one hour and washed with water before use. The cells were plated and incubated at humidity-controlled incubators at 37 °C overnight to allow proper attachment and flattening and fixed with 4% paraformaldehyde (PFA) diluted in 1x Dulbecco’s phosphate-buffered saline (DPBS). We then hybridized the cells with the poly-T primer (Supplementary Table 1) at 1 uM concentration in 2x SSC at 37 °C overnight and washed the unbound and non-specifically bound primers with 30% formamide in 2x SSC at 47 °C for 30 min twice followed by 1x DPBS twice, Ambion nuclease-free water (Thermo Fisher, AM9920) twice, and 2x SSC twice. The poly-T primer was replaced with a poly-T primer containing locked thymidine bases for experiments in Fig. 2. The samples for which reverse transcription was needed were incubated with reverse transcription (RT) buffer composed of 1.875 mM dNTP mix (25 mM each) (Thermo Fisher, R1121), 1x Maxima RT buffer (Thermo Fisher, EP0742), 600 units/ml RNAse inhibitor, murine (NEB, M0314S), 600 units/ml RNasin® Plus RNase Inhibitor (Promega, N2611), 2.5 µM Template Switching Oligo (TSO) (Supplementary Table 1), 10 units/µl Maxima H Minus Reverse Transcriptase (Thermo Fisher, EP0751) at 37 °C overnight and washed with 2x SSC twice and 1x PBS twice to remove excess reagents.

### Photocleavage and ligation of letters for barcode generation

We prepared 2x ligation stock buffer weekly, which was composed of 132 nM Tris-HCl, 20 mM MgCl_2_, and 15% Polyethylene glycol (PEG 8000) at pH 7.6 and stored at room temperature. Immediately before use, we supplemented the stock buffer with 2 mM ATP (Thermo Fisher, R0441) and 2 mM DTT (NEB, 7016L), both of which have only been freeze-thawed once. Samples were washed with 1x ligation buffer twice and incubated with the ligation mixture containing 1x ligation buffer, 0.1 % Triton-X 100 (Sigma-Aldrich, 93443), 0.4 µM splint(s), 0.4 µM letter-representing DNA and 40 U/µl Quick ligase enzyme (NEB, M2200S) for 1 hour at room temperature in dark. We selected letter sequences to have at least 40% GC content with no G or C at the ends of the sequence to support high ligation efficiency^18^. The splints contained sequences complementary to the five bases at the 3’ end of the incoming letter and the ten bases at the 5’ end of the prior letter. The splints are named by using a numbering convention of the incoming letter ID followed by the prior letter ID on the barcode. For example, ligation of L_1_ to L_2_ would be facilitated by the splint12. All letter and splint sequences are shown in Supplementary Table 1 and were ordered from the commercial catalogue of Integrated DNA Technologies (IDT). The chemical structure of the photocleavable linker with the ordering symbol /iSpPC/ in IDT is shown in Supplementary Figure 2. Upon irradiation with UV, this spacer is released leaving a 5’ phosphate group.

Following the ligation, samples were washed with 1x PBS twice, 30% formamide in 2x SSC twice, and nuclease-free water twice to remove excess reagents. Photocleaving for the ligation product was performed using a stand-alone LED light source, 365 nm, or an inverted Olympus IX-71 microscope with a 10x objective coupled to Mightex 1000 series polygon1000G model DMD with a different LED source, 365 nm. We illuminated the desired regions of each sample at an intensity of 3 mW/mm^2^ for 30 seconds with the DMD for experiments in Fig. 3 and 4 or 10 mW/mm^2^ for 30 seconds with the LED for experiments in Fig. 2. We washed the cleaved products with 1x PBS twice and nuclease-free water twice.

### FISH detection of light-controlled barcode letter additions

U2OS cells were prepared as described in the “Sample preparation of cells” section. After fixation, we ligated the entire sample with the PC-containing letter L2 before starting our barcoding ligations such that the whole sample is photocleavable. We then washed the sample in wash buffer composed of 30% formamide in 2x SSC twice and 1x PBS twice. We then incubated the cells with phosphatase buffer composed of 1x rCutsmart buffer (NEB, B6004S), 0.1 % TX-100, 250 U/ml quick CIP enzyme (NEB, M0525S) for 20 min at 37 °C to remove any free 5’ phosphate groups that may have formed due to stray light and washed the samples with 1x PBS once and nuclease-free water once. This ensures that only regions that are photocleaved by light participate in subsequent ligation steps. We illuminated the regions (10% of total cells) of interest using patterned light generated by DMD and proceeded with the ligation of letters. To allow detection of the barcodes by FISH in this experiment, we used letters containing the TGCGAACTGTCCGGCTTTCA or GATCCGATTGGAACCGTCCC sequence downstream (5’) to the PC. This sequence was then detected with a complementary probe conjugated to Atto 565 (FISH probe 1, Supplementary Table 1) or Alexa 647 (FISH probe 2, Supplementary Table 1) with a thiol linkage. After the ligation of letters, we incubated the samples with the FISH probes at a 10 nM final concentration in wash buffer. We removed the excess reagents by incubating the samples in wash buffer for 10 min once and nuclease-free water once. The samples were imaged with an inverted Olympus IX-71 microscope using 560 nm and 647 nm excitation light. We observed that the signal coming from the FISH probes did not go away after photocleaving and extensive washing. This is unlikely to be due to inefficient photocleaving, as that would prevent further ligation, but we observed that the vast majority of the ligation products after the first round were capable of ligation in the second round. It is possible that after photocleaving, the FISH probed labeled photocleavage product remained stuck on the sample. To overcome this problem, we used FISH probes conjugated to fluorophores by a disulfide bond. Following the detection of the first letter, samples were subjected to tris(2-carboxyethyl)phosphine (TCEP) incubation, cleaving the disulfide bond and removing the fluorophores before moving forward. We incubated the samples in wash buffer supplemented with 0.5 M TCEP and blocker sequences at 10 nM (blocker 1 and blocker 2, Supplementary Table 1) for 20 min at room temperature. The blocker sequences are the FISH-probe sequences but without the fluorophores. They were added to bind and block any unhybridized targets of the FISH probes and reduce fluorescent signal carryover into the next round. Following this, the samples were washed with 30% formamide in 2x SSC for 10 minutes and then twice with 2x SSC. Letters ligated in the next round were detected the same way and then treated with phosphatase, as described earlier. We then photocleaved the PC and performed a final round of ligation of the PCR primer.

### Image processing

Images were processed using Fiji (Fiji Is Just ImageJ) and custom scripts. For Fig. 3D the contrast levels of 560 nm (green) and 647 nm (red) channels were adjusted independently but the contrast range was kept identical for the same color in all four regions and across rounds. For Fig. 3F, in each round, we performed two color imaging at 560 nm and 647 nm twice, interleaved by a strong wash to remove the signal in between. The contrast range of each color was adjusted independently but was kept identical for the same color in all regions and across rounds. After the contrast adjustment, the four images per round were merged and colored in red, yellow-green, cyan, and purple-blue in the final figure. Similarly, in Fig. 4B, after stitching all field of views and subtracting the background, the contrast levels of each color channels were adjusted independently but the contrast range was kept identical for the same color. We then merged the two color images to generate the final figure.

### Gaussian fitting to derive barcode size proportions

The data in Fig. 3E and Fig. 3G were derived by fitting the TapeStation electropherogram of the extracted material to a sum of Gaussians. In our Gaussian model, we included components up to one more off-target letter addition than the intended barcode length, where the general form of the fitting function $f$ is given by

$$f\left(l\right)={\sum }_{\mathrm{k}=1}^{\mathrm{R}+1}{\mathrm{A}}_{\mathrm{k}}{e}^{-\frac{{\left(l-{\mu }_{k}\right)}^{2}}{2{\sigma }_{k}^{2}} },$$

where $l$ is the length in base pairs of the cDNA fragment, $R$ is the number of rounds in the experiment, and ${A}_{k}, {\mu }_{k},$ and ${\sigma }_{k}$ are the amplitude, mean, and standard deviation of the $k$ th peak being fitted. While no visible band in the TapeStation gel image or peak on the TapeStation electropherogram was observed for 3-letter barcodes in the sample shown in Fig. 3E, suggesting the absence of such product, we estimated an upper bound for the 3-letter barcode proportion from our Gaussian model. To ensure that the fitting was performed specifically for that extra peak, during the fitting, we restricted ${\mu }_{R+1 }= {\mu }_{R}+20$ and ${\sigma }_{R+1}={\sigma }_{R}$, given that off-target letter addition would result in a barcode that is 20 base pairs longer and the variance of the measured size of each species detected on TapeStation should be similar. In Fig. 3G, a peak corresponding to a species with one off-target letter addition was visible; we used the observed intensity of this peak and did not constrain any parameters.

### Determination of on-target and off-target letter addition probabilities in spatially resolved barcoding experiments

Let *n*_*L*_ denote the number of unique letters used per round and *R* the number of rounds. Each round consists of two consecutive letter-addition reactions, each associated with a distinct illumination pattern. In a given reaction, the targeted cell receives the corresponding letter with probability, *d*. In untargeted reactions, the cell may still acquire a letter with probability, *c*, due to off-target photocleaving and ligation. Then the probability of generating a barcode of length *N* is given by the following formula where *k* is the number of on-target letter addition events:

$$P\left(L=N\right)={\sum }_{k=\mathrm{max}\left(0, N-\left({n}_{L}-1\right)R\right)}^{\mathrm{min}(R, N)}\left(\genfrac{}{}{0pt}{}{R}{k}\right){d}^{k}{\left(1-d\right)}^{R-k}\left(\genfrac{}{}{0pt}{}{{(\mathrm{n}}_{\mathrm{L}}-1)R}{N-k}\right){c}^{N-k}{\left(1-c\right)}^{{(n}_{L}-1)R-(N-k)}.$$

The maximum number of on-target letter additions is equal to number of rounds, *R.* Therefore, we impose an upper limit for *k* in the formula considering barcodes longer than the maximum number of on-target events, *R*, in which case the remaining letters are added through off-target events. Similarly, the maximum number of off-target events is equal to *(n*_*L*_*-1)R.* Therefore, we impose a lower limit for *k*, when the barcode length is longer than the maximum number of off-target events, *(n*_*L*_*-1)R*, in which circumstance the remaining letters, *N*-*(n*_*L*_*-1)R*, are generated through on-target events*.*

Using this formula, and the fraction of barcodes with different length, we determined the on-target letter addition rate* d* and off-target letter addition rate* c* from results shown in Fig. 3D-G.

### FISH detection of light-controlled barcodes in the co-culture experiment

In these experiments, we perform FISH labeling in two steps; the first step is with an adaptor probe that contains both a target sequence complementary to a barcode letter and a readout sequence that can be detected by a complementary, fluorescently labeled readout probe. We used adaptor probes complementary to part of L_1_L_3_ (Barcode 1 adaptor probe sequence, Supplementary Table 1, Supplementary Figure 4) and L_2_L_4_ (Barcode 2 adaptor probe sequence, Supplementary Table 1, Supplementary Figure 4) flanked by readout sequences to detect the barcodes in the sample. After barcoding was completed, we incubated the samples with the adaptor probes at 0.1 µM concentration diluted in wash buffer composed of 30% formamide, 2x SSC, for 20 min at room temperature followed by a 20 min wash step with the wash buffer to remove the non-specifically bound and excess reagents. We then incubated the samples with the readout probes (readout probe 1 or 2, Supplementary Table 1) at 10 nM final concentration diluted in wash buffer for 20 minutes and washed the excess reagents with the wash buffer for 10 minutes. All incubation and wash steps were performed in the dark. For quantification, images are thresholded to remove background and analyzed using the “Analyze particles” module in ImageJ to determine the number of fluorescently labeled cells.

### Extraction and detection of barcoded primer or cDNA for barcode-size measurements

To extract the primer or cDNA from the samples, we heated the samples in 50 ul water supplemented with 0.1 % Triton-X 100 to 93 °C for 4 min and immediately aspirated the solution into an Eppendorf tube and left it at room temperature for several minutes for it to cool down. The DNA ScreenTape assays used for Agilent TapeStation system are designed to analyze double stranded products. Therefore, for Fig. 2B and C, once we extracted the product we generated either a full double strand by performing one PCR cycle using only the reverse primer with Seqamp DNA polymerase (Takara Bio., 638504) in SeqAmp CB PCR buffer (Takara Bio., 638526) according to the manufacturer’s instructions (for two replicates) or a partial double strand by annealing the extracted product with a strand complementary to a short portion of the primer sequence, generating a chimera containing single and double-stranded regions (for one replicate). In addition, for this latter replicate only, we used a different primer that leads to a longer product (Supplementary Table S1). For Fig. 2D and 3D, we performed 5-10 rounds of qPCR using both forward and reverse primers. We then purified the PCR product using Monarch PCR & DNA cleanup kit (New England Biolabs, T1030L) according to the manufacturer’s instructions. The purified product was run in a High Sensitivity D1000 ScreenTape (Agilent, 5067-5584) using the Agilent 2200 TapeStation system. We note that the single/double-stranded chimeric product generated in one replicate of Fig. 2B and C runs at a different migration speed compared to full double-stranded product in the remaining two replicates due to the differences in their lengths and structures. While there was a difference in the final position of the products due one replicate being partially double-stranded, the proportions as well as the relative differences in size of species with consecutive letter additions are consistent with each other for all replicates.

### Library preparation and RNA sequencing analysis

For samples used for sequencing, we ligated SmartSeq3 sequences (Supplementary Table 1) to the barcoded cDNA. We extracted the cDNA similarly as described in the previous section and amplified the extracted material using long-distance (LD) PCR with Seqamp DNA polymerase according to the instructions outlined in the Takara Smartseq V4 kit using a forward sequence complementary to Smart seq3 and a reverse primer complementary to the TSO sequence. The amplified product was purified using Ampure XP beads at a 0.8x v/v ratio. We used the Nextera XT DNA Library Preparation Kit (Illumina, FC-131-1024) for tagmentation with a few modifications. First, we amplified the tagmented products with our custom forward primer (Illumina Read 1 + SmartSeq3 sequences) and Illumina Read 2 reverse primer (Supplementary Table 1) for 5’ enrichment. Following the purification of the PCR product using Ampure XP beads at 0.8x v/v ratio, we performed another PCR round to add the Illumina P5, i5, P7, and i7 sequences for downstream sequencing and purified the products using Ampure XP beads at 0.8x v/v ratio. Sequencing was performed in the Miseq system using the MiSeq Reagent Kit v3 (150-cycle) (Illumina, MS-102-3001) with 150 paired-end reads. The FASTQ output was filtered for reads with the appropriate barcode sequence in the Read 1 file with a custom python script. The corresponding filtered Read 2 file was mapped onto the mouse genome (GRCm39) and human genome (GRCh38.p14) separately using STAR^19^ as single-ended reads in quantMode and keeping maps with only one unique match. The Read 1 file was not used for STAR to prevent the barcode sequences from being used in the genome alignment. This generated a BAM file aligned to the transcriptome. The mRNA transcript counts were enumerated from the output BAM file using RSEM^20^ with the appropriate mouse or human transcriptome references using annotations GENCODE vM33 for Mouse and GENCODE v44 for Human.

## Supplementary Information

Supplementary Information.

## Data Availability

The sequencing data were deposited into sequence read archive (SRA) database under accession number PRJNA1236399 and are available at the following URL: https://www.ncbi.nlm.nih.gov/sra/PRJNA1236399. Other data that support the findings of this paper are available from the corresponding authors upon request.

## Code availability

Software used to analyze the data is available from the corresponding authors upon request.
