# Supplementary material for: Multiplexed optical barcoding and sequencing for spatial omics
Source: Sci Rep. 2026 Mar 18;16:14086. doi: 10.1038/s41598-026-41186-y (PMC13136315; doi:10.1038/s41598-026-41186-y)
Supplement: Supplementary file 1 — Supplementary Information. [file 41598_2026_41186_MOESM1_ESM.pdf]

Supplementary information for

Multiplexed optical barcoding and sequencing for spatial omics

Aditya Venkatramani, Didar Ciftci, Khanh Pham, Limor Cohen, Leonardo Sepulveda, Christopher Li, Xiaowei Zhuang

Supplementary information includes

Supplementary Figures 1 to 4

Supplementary Table 1

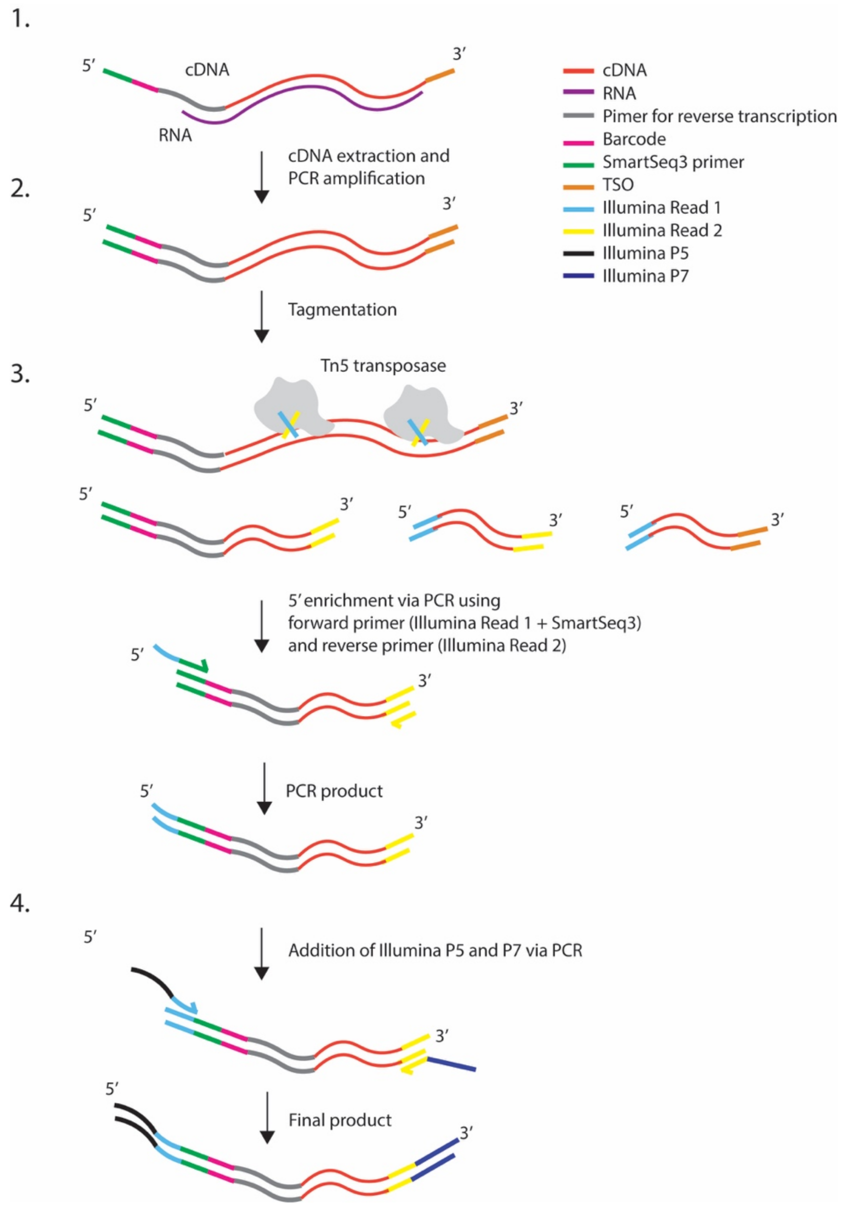

Supplementary Figure 1. Schematic of library preparation for sequencing with 5’ enrichment. Step 1. We start with reverse transcribed cDNA that has a template-switching oligonucleotide (TSO) sequence in the 3’ end and is barcoded in the 5’ end with light-controlled ligations of barcode letters followed by one additional ligation step to add a SmartSeq3 sequence. Step 2. We perform PCR to amplify the barcoded cDNA. Step 3. The amplified product is tagmented with Tn5 transposase to add the Illumina Read 1 and Illumina Read 2 primers, using Illumina Nextera XT kit, and selected for the 5’-end product by performing PCR on the tagmented product with the forward primer (SmartSeq3 + Illumina Read 1 primer) and reverse primer (Illumina Read 2 primer). Step 4. The 5’ enriched product is extended to add the Illumina P5 sequence on the 5’ end and the Illumina P7 sequence on the 3’ end by PCR.

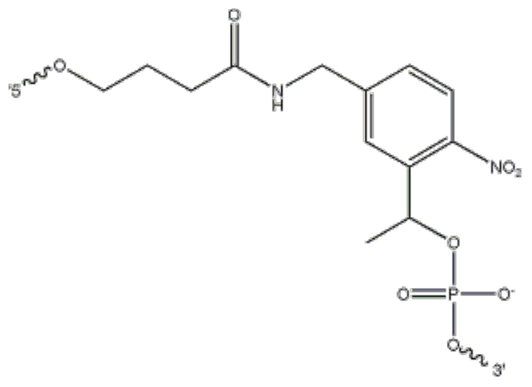

Supplementary Figure 2. Chemical structure of /iSpPC/ photocleavable spacer used in letter oligonucleotides. Upon irradiation with UV light, this molecule is released, leaving the 3' connected end with a 5' phosphate group.

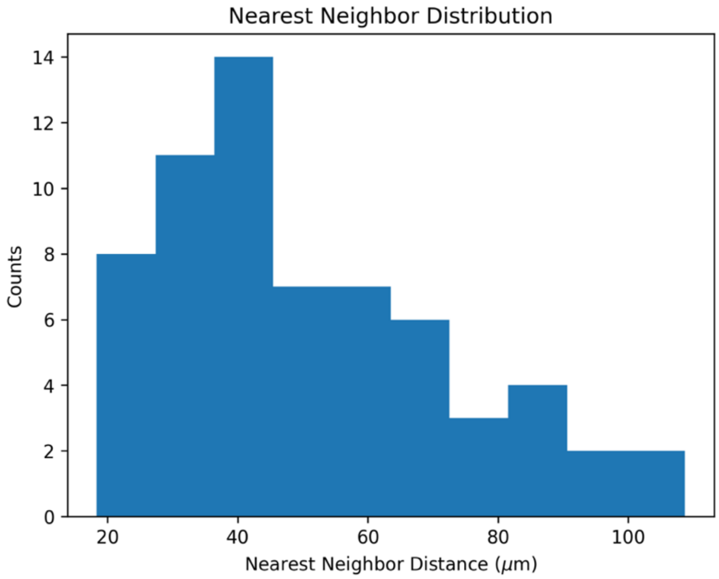

Supplementary Figure 3. Nearest-neighbor distribution of the 64 cells barcoded in Figure 3F-G. For a single cell we calculate the distance between that cell and every other cell and then take the minimum distance as the nearest-neighbor distance for that particular cell. We then repeat the calculation for every cell and plot the resulting distribution. The median value of the nearest-neighbor distances was 44 μm.

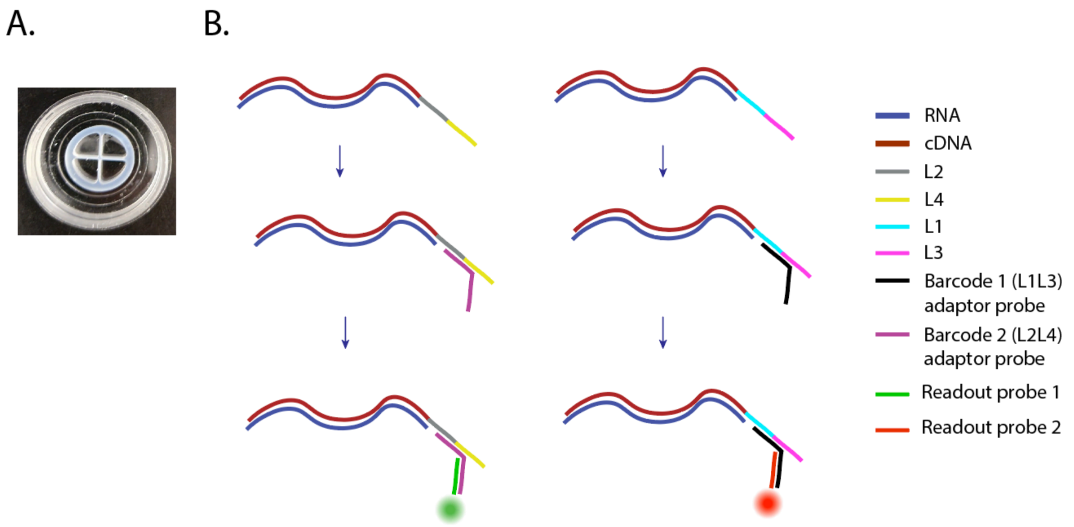

Supplementary Figure 4. Plating of two different cell species for co-culture experiment and detection of spatial barcodes using FISH. **A.** Photo of Ibidi Culture-Insert 4 Well in µ-Dish 35 mm where the cells are plated. The two different species of cells (MEF and U2OS) were plated and fixed in two adjacent wells. The silicone insert creating the wells was removed before barcoding. **B.** Schematic of FISH detection of barcodes in the co-culture experiment. The two different regions have distinct barcodes comprising two letters in each barcode (L2L4 and L1L3). Adaptor probes complementary to part of each letter in the barcode, flanked by a readout sequence, are hybridized to the barcodes. The adaptor probes are detected using fluorescent readout probes.

Supplementary Table 1. Sequences of primers, letters, splints, adaptor probes, FISH probes (readout probes) used in this study. All oligonucleotides are purchased in lyophilized form from Integrated DNA Technologies (IDT).

poly-T primer for reverse transcription	/5Phos/AGAGA ATGGA TAGGT TGTGT NNNNNNNNNNNNNNNNNNNN TTTTTTTTTTTTTTTTTTTTTTTTTTTTTT
poly-T primer for barcode size detection (used for two replicates in Figure 2B-2C)	/5Phos/ AGAGA ATGGA TAGGT TGTGT AATCAGCCATACCACATTTG TT +TT +TT +TT +TT +TT +TT +TT +TT +TT +TT +TTT
poly-T primer for barcode size detection (used for one additional replicate in Figure 2B-2C)	/5Phos/ AGAGA ATGGA TAGGT TGTGT TT +TT +TT +TT +TT +TT +TT +TT +TT +TT +TT +TTT AATCAGCCATACCACATTTG
TSO	AAGCAGTGGTATCAACGCAGAGTACATrGrG+G
Letter 1 (L1)	TTTTTTTTTTTTTTTTTTTT/iSpPC/AGAGAATGGATAGGTTGTG T
Letter 2 (L2)	TTTTTTTTTTTTTTTTTTTT/iSpPC/ATGGAAGAGATGTGTTAGG T
Letter 3 (L3)	TTTTTTTTTTTTTTTTTTTT/iSpPC/TAGGTTGTGTAGAGAATGG A
Letter 4 (L4)	TTTTTTTTTTTTTTTTTTTT/iSpPC/TGTGTTAGGTATGGAAGAG A
Letter 1 (L1) for fluorescent detection	GAT CCG ATT GGA ACC GTC CC/iSpPC/AGAGA ATGGA TAGGT TGTGT
Letter 2 (L2) for fluorescent detection	TGC GAA CTG TCC GGC TTT CA/iSpPC/ATGGA AGAGA TGTGT TAGGT
Letter 3 (L1) for fluorescent detection	TGC GAA CTG TCC GGC TTT CA /iSpPC/TAGGT TGTGT AGAGA ATGGA
Letter 4 (L2) for fluorescent detection	GAT CCG ATT GGA ACC GTC CC /iSpPC/TGTGT TAGGT ATGGA

	AGAGA
Splint12	TCTCTTCCATACACA
Splint21	TCCATTCTCTACCTA
Splint31	TCCATTCTCTTCCAT
Splint42	TCTCTTCCATTCTCT
SmartSeq3 forward	ACGAGCATCAGCAGCATACGA
Reverse primer for library preparation	AAGCAGTGGTATCAACGCAGAGT
Barcode 1 adaptor probe sequence	TCC ATT CTC TTC CAT TCT CTT GCG AAC TGT CCG GCT TTC A
Barcode 2 adaptor probe sequence	TCTCTTCCATTCTCTTCCATGATCCGATTGGAACCGTCCC
FISH probe 1 (readout probe 1)	/5ATTO565N//iThioMC6-D/T GAA AGC CGG ACA GTT CGC A
FISH probe 2 (readout probe 2)	/5Alex647N//iThioMC6-D/G GGA CGG TTC CAA TCG GAT C
Blocker 1	T GAA AGC CGG ACA GTT CGC A
Blocker 2	G GGA CGG TTC CAA TCG GAT C
Reverse primer for second strand synthesis	CAAATGTGGTATGGCTGATT

Forward primer for 5’ enrichment	TCGTCGGCAGCGTCAGATGTGTATAAGAGACAG ACGAGCATCAGCAGCATACGA
Reverse primer for 5’ enrichment	GTCTCGTGGGCTCGGAGATGTGTATAAGAGACAG
